# Knowledge, perceptions and practices on healthcare waste management and associated occupational health hazards among healthcare professionals in the Colombo District, Sri Lanka: a cross-sectional study

**DOI:** 10.3389/fpubh.2023.1215648

**Published:** 2023-12-27

**Authors:** Lahiru Udayanga, Loganatahan Sahana, Ayesha Perera, Koshila Ranasinghe, Tharaka Ranathunge

**Affiliations:** ^1^Department of Biosystems Engineering, Faculty of Agriculture and Plantation Management, Makadura, Wayamba University of Sri Lanka, Kuliyapitiya, Sri Lanka; ^2^Durdans Hospital, Colombo, Sri Lanka; ^3^Department of Zoology and Environmental Management, Faculty of Science, University of Kelaniya, Colombo, Sri Lanka; ^4^Department of Zoology, Faculty of Faculty of Science, Eastern University, Chenkalady, Sri Lanka

**Keywords:** knowledge and attitudes, healthcare waste, health hazards, occupational health, Sri Lanka

## Abstract

**Background:**

Proper Healthcare Waste (HW) management is directly influenced by the knowledge and attitudes of Healthcare Professionals (HCP). However, studies that characterize the knowledge and practices of HCP on HW management are limited in Sri Lanka. This study was conducted to characterize the knowledge, perceptions and practices of HCP on the management of HW and to determine the risk factors influencing HW related occupational health hazards in the Colombo District of Sri Lanka.

**Methods:**

A total of 407 HCP were recruited as the study population from selected hospitals in the Colombo District. Information on socio-demographic factors, knowledge, attitudes and practices on HW management were gathered using an interviewer-administrated questionnaire. The Binary Logistic Regression (BLR) was used to determine the socio-economic risk factors associated with the occurrence of HW related health issues among the respondents.

**Results:**

The majority of respondents were characterized with a high knowledge level (76.9%) and positive attitudes (53.8%) on HW management. Incineration (82.6%) was recognized as the most widely used HW treatment method. Personal Protective Equipment (PPE) was used at a satisfactory level (85.5%), while liquid waste treatment was limited (57.5%). The occupational designation, level of training received in HW management, professional experience, vaccination status for tetanus, degree of knowledge and attitudes on HW management were recognized as significant risk factors (*p* < 0.05) associated with the occurrence of HW related occupational hazards.

**Conclusion:**

Even though, the treatment of HW was satisfactory, strengthening the existing mechanisms for monitoring of HW management, provisioning more resources and organizing training and awareness programmes on HW management for HCP are recommended.

## Introduction

Hospitals are one of the dynamic institutions used by general public without any distinction of age, sex, race, or religion (1). Health services eventually produce waste that can itself be harmful to environment and human health, while attending to health issues and treating patients (2). Any waste created during the diagnosis, treatment, or immunization process, or in research activities related thereto, or health camps are considered as Healthcare Waste (HW) or biomedical waste (3). Generation of enormous amounts of HW has been recognized as a major concern in many countries. The reckless and indiscriminate disposal of HW could lead to unpleasant odour, proliferation of insects and rodents, incidence of epidemics such as typhoid, cholera, and hepatitis (4–6).

Around 85% of HW is currently non-hazardous, while 10% is infectious and 5% is non-infectious but hazardous (7–9). Another study has reported that approximately 15 to 35% of HW generated in Bangladesh is infectious (9). Discarded blood, removed organs, surgical waste, sharps, undesired microbiological cultures, and expired medication etc. are examples for infectious waste (10). Therefore, storage, disposal and proper treatment of HW have become a major concern for both the medical and general communities (7). Therefore, Healthcare Professionals (HCP) with adequate knowledge, experience, and ability are required to handle HW and to ensure proper management of HW (11).

According to the World Health Organization (WHO), HW has been categorized into eight categories as, general waste, pathological waste, radioactive waste, chemical waste, infectious and possibly contagious waste, sharps, pharmaceuticals, and pressurized containers (12). Meanwhile, the Basel Convention has suggested five broad categories of HW as, non-hazardous HW, HW with special attention, infectious and highly infectious HW, radioactive HW and other hazardous waste (7, 13). Composition and generation rates of HW often vary between developed and developing nations. Generally developing countries denote lower HW generation rates than developed countries. However, HW generation rates are increasing significantly at the global level, mainly due to improved access to healthcare services and increasing aging population (14).

Even though, many countries are having different legislations and policies related to HW management, many developing nations are denoting poor HW handling practices. This directly reflects lack of adequate attention on HW management (15). At the hospital level, the effectiveness of HW management relies on a committed waste management team, good management practices, proper preparation, sound organization, robust legislation, sufficient support, and active participation of trained employees. National regulatory structure, internal management systems, training programmes, and the use of suitable disposal techniques, are crucial to ensure proper HW management (10, 16).

Health-care systems in Sri Lanka are progressing. However, the increasing population and their requirements have resulted HW management to become a complicated and demanding concern. The national strategy on HW management in Sri Lanka enacted in 2001, has demanded all healthcare providing facilities to develop specialized HW management plans under the supervision of the Provincial Department of Health Services (PDHS). Further, a colour code system for segregation of HW into seven categories was developed in 2006 (17). However, enormous quantities of HW generation, negligence and/or inadequate qualifications of personnel in charge of HW management have caused a poor performance of HW management in Sri Lanka (18). The absence of strategies and plans for correct and effective HW collection, transportation, and treatment, along with higher expenses associated with HW management, have further aggravated this issue (18, 19).

If handled improperly, HW may lead into numerous occupational hazards among HCP (10). According to Athapattu et al. (18), even though many government hospitals in Colombo are conscious of the dangers/health consequences of HW, environmental impacts or contaminations that may be caused by poor HW management remains neglected. Further, many gaps in HW management have been observed in Sri Lanka (18, 19). However, comprehensive studies that characterize the knowledge and practices of HCP on HW management in Sri Lanka are limited. Therefore, this study aimed to characterize the knowledge, perceptions and practices of HCP on the management of HW and to determine the risk factors associated with the occupational health hazards arising from poor HW management in government and private hospitals in the Colombo District of Sri Lanka.

## Methodology

### Study design and sampling

This analytical cross-sectional study, considered seven major government (namely, Sri Jayawardenapura Hospital, Sir John Kothalawela Defense Hospital, Dr. Nevil Fernando Teaching Hospital and Maligawatta Primary Care Unit) and private hospitals (Durdans Hospital, Kings Hospital and Nawaloka Hospital) in the Colombo District. These depict different healthcare service levels in the Sri Lankan health system (primary, secondary and tertiary). The Lwanga and Lemeshow equation (20) was used to estimate the sample size as 385 HCP, at a precision of 5%, while the population proportion was set as 0.5 (50%). During the fieldwork, the sample size was increased up to 407 HCP. The respondents were recruited using the stratified random sampling technique, while the nature of the hospitals (government or private) and the health care service levels (primary, secondary and tertiary) were considered as strata. The respondents were recruited after acquiring the informed written consent and HCP who were not willing to cooperate in the study due to one or more reasons such as personal reasons or their opinion that it is not worthwhile participating in our survey, were not considered for the survey. On such occasions, the sample size was achieved by randomly selecting new respondents with consent to participate for the study.

### Data collection

An interviewer administrated pre-tested questionnaire was used for data collection. The questionnaire consisted of four broad categories as mentioned below.

*Section A:* Basic socio-demographic information of the participants such as age, gender, ethnicity, education level, marital status, official designation, years of service and vaccination status for Hepatitis B and tetanus etc. were collected under this section.

*Section B:* A set of 14 questions comprised of ten (10) Multiple-Choice Questions (MCQ) and four (04) dichotomous questions were used to assess the knowledge of respondents on the definition of HW, their categories, risks associated with HW, appropriate disposal and storage methods of HW etc.

*Section C:* Practices on HW management such as practicing of appropriate collection and storage methods for different types of HW, use of Personal Protective Equipment [PPE], different disposal methods used for HW and practicing of contingency plans etc. were evaluated under this section. In addition, whether the respondent has experienced any adverse health impacts from HW or not was inquired.

*Section D:* A set of fifteen (15) Likert scale statements were used to assess the attitudes of respondents on HW management. These statements covered perceptions on the risks associated with HW, importance of using appropriate collection and disposal methods for HW and satisfaction level on the support provided by administrative staff for HW management etc.

### Data analysis

All collected data were verified for completeness and entered into Microsoft Access® data sheets. Discrepant data were checked against original data forms. Sub index of scores were calculated for knowledge and attitudes. In case of knowledge, percentage of correct answers provided for the fourteen (14) knowledge-based questions included under Section B were used to calculate the knowledge sub-index. The knowledge level on HW of the participants were classified in to three categories as “Good” (> 66.67%), “Moderate” (33.34 to 66.66%) and “Poor” (< 33.33%) based on a percentage score obtained for Section B, as suggested by Udayanga et al. (21). For the attitudes, each statement was ranked on a five-point Likert scale and the overall attitude sub-index was calculated as shown in Eq. 1.


(1) $$\mathrm{Attitude}\;\mathrm{Subindex}=\frac{\Sigma W}{\left(A*N\right)\;}$$

Where, W is the rank provided by the respondent for each statement, A is the maximum rank allowed (5 points) for each statement and N is the total number of statements (15) considered under the Section D. After calculation, attitudes sub-index was classified in to three categories as “Good” (> 66.67%), “Moderate” (33.34 to 66.66%) and “Poor” (< 33.33%) based on the score obtained for Section D (21). The Binary Logistic Regression (BLR) with forward step-wise variable selection method was used to determine the socio-economic risk factors that could lead into HW related occupational hazards among the respondents. The fact whether respondents have experienced any adverse health impacts from HW or not was used as the response variable. Other, socio-demographic variables (gender, age, ethnicity, marital status and educational level etc.), occupation related variables (nature of the employed hospital, professional designation, experience, length of the duty period and vaccination status for tetanus and hepatitis B), knowledge level on biomedical waste management and attitude level on biomedical waste management etc. were used as the predictor variables. IBM SPSS Statistics software package (Version 23) was used to analyze the data.

## Results

### Socio-demographic factors of the study population

The socio-demographic details of the respondents are shown in Table 1. Female respondents accounted for the majority (56.8%). Respondents belonging to the 31–40 years old age group (36.4%) dominated the sample, followed by the 20–30 years old group (32.7%). Completion of a diploma or degree was the highest educational qualification of the majority (46.7%). A higher fraction of respondents (31.4%) was working as Labourers (31.4%) or Attendants (9.1%), followed by Nursing Officers (28.7%) and Medical Officers (20.9%). Around 51.1% of the study population was employed at private hospitals, while the remaining (48.9%) were employed at government hospitals. Among the government hospitals, the highest fraction was employed at secondary hospitals (21.9%), followed by tertiary hospitals (21.1%).

**Table 1:** Socio-demographic factors of the study population.

Parameter		Total respondents	
		*n*	%
Gender	Male	176	43.2
	Female	231	56.8
Age (Years)	20–30	133	32.7
	31–40	148	36.4
	41–50	95	23.3
	>50	31	7.6
Ethnicity	Buddhism	294	72.2
	Hinduism	70	17.2
	Islam	16	3.9
	Christianity	27	6.6
Marital status	Married	240	59.0
	Unmarried	163	40.0
	Separated/Widowed	4	1.0
Educational level	Illiterate/Primary	104	25.6
	O/L	57	14.0
	A/L	9	2.2
	Diploma/Degree	190	46.7
	Post-Graduate/MD	47	11.5
Type of hospital, your employed at?	Primary (Government)	24	5.9
	Secondary (Government)	89	21.9
	Tertiary (Government)	86	21.1
	Private	208	51.1
Designation	Medical Officers	85	20.9
	Nursing Officers	117	28.7
	Paramedical Staffs	37	9.1
	Attendants	40	9.8
	Labourers	128	31.4
Length of your duty per day (Hours)	< 10	154	37.8
	10 to 15	174	42.8
	15 to 20	18	4.4
	> 20	61	15.0
Years of service	Less than 3 Years	65	16.0
	3 to 5 Years	109	26.8
	6 to 10 Years	1.6	26.0
	More than 10 Years	127	31.2
Have you ever received any training on medical waste management programmed?	Yes	271	66.6
	No	136	33.4
Prominent source of information about medical waste management?	Hospital	352	86.5
	Collage	23	5.7
	Authorized source	11	2.7
	Others	21	5.2
Have you been vaccinated for Hepatitis B?	Yes	372	91.4
	No	35	8.6
Have you been vaccinated for Tetanus?	Yes	291	71.5
	No	116	28.5
Have you ever encountered any sharp/needle stick injury in the last 12 months?	Yes	56	13.8
	No	351	86.2
Ever been affected due to poor Healthcare waste management?	Yes	347	85.3
	No	60	14.7

A notable fraction of the study population (31.2%) was having more than 10 years of experience in the health sector. Around 42.8% of respondents were serving 10–15 h per day, followed by another 37.8% serving <10 h per day. Interestingly, a notable faction of respondents (66.6%) had received a formal training on HW management. The majority of employees were vaccinated for Hepatitis B (91.4%) and Tetanus (71.5%) by the time of data collection.

### Knowledge on biomedical waste management

Around 65.4% of respondents were aware of HW management. Even though, around 91.6% of the respondents claimed to be familiar with the colour coding system for segregation of HW, less than 70% of them were knowing that yellow-coloured bins are used for infectious waste (69.5%) and black coloured bins are used for general waste (66.1%). The majority of them were aware of the broad categories for classification of HW used in Sri Lanka, while only 37.1% were aware that body fluids, body parts and fecal matter are belonging to pathological waste (Table 2). However, around 87.5% of respondents were familiar with the internationally accepted symbol for biohazards.

**Table 2:** Knowledge on biomedical waste management among respondents.

Parameter		Total respondents	
		*n*	%
Biomedical waste management is,	Activities and actions taken to manage healthcare waste from its inception to final disposal	266	65.4
	Collection of healthcare waste from one central place	71	17.4
	Disposal of collected healthcare waste	50	12.3
	Generation of healthcare waste	20	4.9
Do you know about the colour coding system for segregation of medical waste?	Yes	373	91.6
	No	34	8.4
What type of medical waste should be disposed into a yellow-coloured waste bin?	General waste	106	26.0
	Infectious waste	283	69.5
	Plastic waste	15	3.7
	Radioactive waste	3	0.7
What type of medical waste should be disposed into a black coloured waste bin?	General waste	269	66.1
	Infectious waste	50	12.3
	Plastic waste	78	19.2
	Radioactive waste	10	2.5
Which of the following is the internationally accepted symbol for biohazards?	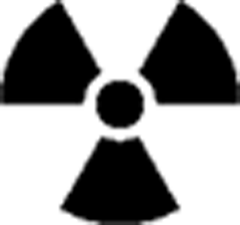	25	6.1
	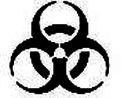	356	87.5
	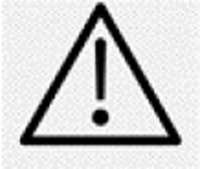	12	2.9
	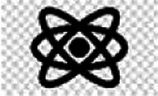	14	3.4
What is the maximum time of storage for infectious medical wastes before being treated or disposed of?	24 h	294	72.2
	48 h	107	26.3
	72 h	3	0.7
	> 72 h	3	0.7
What is the maximum extent for a waste container containing needle and/or sharp materials should be filled?	½ full	49	12.0
	¼ full	9	2.2
	¾ full	276	67.8
	Full	73	17.9
Paper, Food, Plastic, Bottles belong to	General waste	355	87.2
	Infectious waste	27	6.6
	Pathological waste	18	4.4
	I do not know	7	1.7
Soiled cotton wool, Swab, Gloves belong to	General waste	31	7.6
	Infectious waste	370	90.9
	Radioactive waste	1	0.2
	I do not know	5	1.2
Needles, Scalpels, Syringes belong to	Infectious waste	18	4.4
	Pathological waste	21	5.2
	Sharps waste	362	88.9
	I do not know	6	1.5
Body fluids, Body parts, Fecal matter belong to	General waste	6	1.5
	Infectious waste	246	60.4
	Pathological waste	151	37.1
	I do not know	4	1.0
All types of waste generated at hospitals or laboratories are biological hazardous	Yes	247	60.7
	No	160	39.3
A health institution can produce liquid healthcare waste	Yes	328	80.6
	No	79	19.4
Certain diseases could spread due to improper waste management	Yes	389	95.6
	No	18	4.4

**Figure 1:**
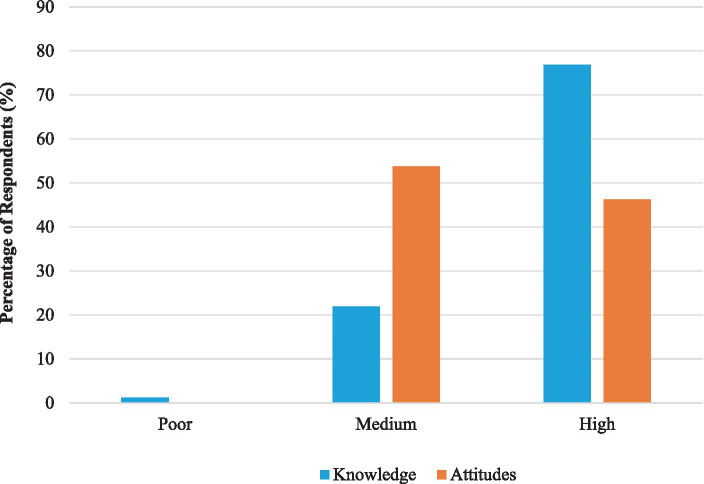
Level of knowledge and attitude scores of health care professionals on healthcare waste management.

Around 72.2% of respondents knew that the maximum time of storage for HW before being treated or disposed is 24 h. Further, the majority of respondents were aware on the risks associated with HW such as, health institutions can produce liquid healthcare waste (80.6%), certain diseases could spread due to HW (95.6%) and expired drugs can cause negative health effects (82.3%). However, only 41.8% of participants were aware of the fact that sharp waste can be dangerous to human health. Meanwhile, good practices related to HW management such as, wearing PPE during handling of HW (90.9%) and disinfection of HW would decrease the risk of infection transmission (82.1%), necessity of closing HW containers during transport (88.5%) and importance of HW incineration for disposal (76.4%) were familiar to the majority (Table 2). Further, a higher fraction of respondents was aware that HW waiting for treatment and/or disposal should be secured (84.3%), any needle-stick injury during handling of HW should be reported and attended by medical staff (81.1%) and hospital incinerator is a source of air pollution (80.3%). Based on the overall knowledge score, 76.9% of HCP were characterized with a high knowledge level on HW management, while 21.9% had a moderate knowledge (Figure 1).

### Attitudes on biomedical waste management

The majority of respondents were perceiving that proper HW management (91.1%) is important and strictly necessary (90.2%), along with segregation of HW at the source (76.9%). Only 50.8% of respondents believed that following HW management practices are an unnecessary extra work burden, while 36.1% perceived that labelling HW containers is important for proper HW management (Table 3). Meanwhile, 65.1% of respondents considered needle stick injuries as a major health concern, while 67.1% perceived that proper HW disposal is important in preventing transmission of infections. Only 37.6% of respondents believed that proper disposal of HW should totally be the responsibility of the government, while the majority perceived that it’s a responsibility of all HCP (67.1%).

**Table 3:** Attitudes of health care professionals on biomedical waste management.

Statement	Percentage response (%)				
	Strongly agree	Agree	Neutral	Disagree	Strongly disagree
Health care waste management is important.	67.8	23.3	6.4	0.5	2.0
Strict implementation is necessary for proper healthcare waste management.	58.0	32.2	6.9	1.5	1.5
Medical waste be segregated at the point of generation.	42.5	34.4	18.9	2.2	2.0
Medical waste management practices are unnecessary extra work burden.	10.3	21.9	17.0	21.6	29.2
Labelling medical waste containers do not add any value to waste management.	16.2	19.9	19.4	21.1	23.3
Needle stick injury is a major health concern.	37.6	27.5	23.8	9.1	2.0
Proper medical waste disposal is important to prevent transmission of infections.	39.1	28.0	23.8	5.7	3.4
Proper disposal of medical waste should totally be the responsibility of the government.	22.1	15.5	20.1	22.4	19.9
Safe medical waste management is a responsibility of all healthcare staff.	41.5	25.6	21.4	8.1	3.4
Residences located near hospitals are suffering more health effects from improper handling of medical waste.	22.6	28.0	25.3	15.7	8.4
I/my institution follow procedures for collecting/handling of waste.	28.5	34.9	27.0	6.4	3.2
The level of medical waste management practices at my institution is satisfactory.	31.2	32.7	26.8	4.9	4.4
Attention of administrative officers on medical waste management at my institution is low.	15.7	19.9	28.0	22.4	14.0
My knowledge on medical waste management practices is satisfactory.	29.2	31.2	29.0	7.1	3.4
Current trainings that I have attained on medical waste management are satisfactory.	23.1	28.5	34.2	9.6	4.7

Interestingly, a notable fraction of HCP was satisfied with the adequacy of existing HW management practices (63.9%) and procedures followed during handling of HW (63.4%) at their institutions. Around 50.6% of the respondents believed that residences located near hospitals were suffering from health effects arising from poor HW management. Interestingly, around 35.6% of HCP were believing that attention of administrative officers on HW management at their workplaces is low. Around 61.4% were satisfied with their knowledge level on HW management practices, while only 51.6% of respondents were contented with their level of training. Meanwhile, around 53.8% of the respondents had a moderate level of attitudes on HW management, while 46.2% had a high level of attitudes (Figure 1).

### Practices related to biomedical waste management

Incineration was recognized as the most common treatment method used for HW management in majority of hospitals (82.6%), followed by sterilization (54.8%) and chemical treatment (38.8%), as shown in S1 Table. Around 58.7% of respondents claimed that open air burning on ground was practiced as the most common incineration method, followed by low temperature incineration (24.3%). However, the incinerator was fenced in many facilities to prevent unauthorized access (72.2%). A high number of respondents were using PPE during handling of HW (85.5%). Gloves (98.5%), boots (84.3%) and aprons (74.6%) were used mostly, while goggles (36.8%) were used limitedly. Less than two third of the respondents were using these PPE always (60.0%) during handling of HW, while a notable fraction was using PPE occasionally (14.3%) or rarely (16.5%).

A notable faction of HCP reported that HW was collected thrice (38.6%) or twice (37.6%) per day, mostly using closed lid containers (91.1%). Human anatomical waste/animal wastes were mainly incinerated (71.0%), along with microbiology/biotechnological waste (62.1%). In case of sharp wastes, incineration (59.2%), microwaving (27.2%) and deep burial (26.0%) were used for treatment. The common disposal method for expiry drugs was returning to the national medical stores (48.2%). However, only a limited fraction of healthcare facilities were treating liquid/chemical waste prior being released in to drains (57.5%). Relevant staff handling HW was mostly provided with routine trainings on HW management (40.5%), or when requested (29.7%). However, most of the HCP disclosed that proper segregation of waste into different categories (88.5%), disinfection of waste collection bins on a daily basis (61.2%), use of puncture proof boxes for collection of sharps waste (91.2%) are being done at a satisfactory level (Supplementary Table S1), along with disposal of sharps containers or needle-destroyers adequately (86.0%).

However, used syringe needles were being collected without recapping (79.9%), while infectious HW was collected from service areas within 24 h (78.9%). Plastic bags or specialized reusable containers (74.0%) were being used for non-sharp infectious waste materials. Many healthcare facilities had plans for treatment and disposal of hazardous chemicals, pharmaceuticals and radioactive waste (68.3%). Only 48.2% of respondents were disposing the incineration ash of waste into a municipal landfill or open dumping site. However, 64.4% of respondents suggested that existing storage facilities for HW were adequate and meeting standard guidelines. Meanwhile, only around half of the healthcare facilities were treating laboratory cultures and stock of infectious agents (53.3%) and liquid waste (57.7%) prior disposal. Less than two third of the respondents suggested that treatment facilities undergo regular inspection and periodic maintenance (57.5%) and are equipped with standard treatment technology such as autoclave-shredder, integrated steam treatment system, or microwave unit (60.2%). Interestingly, scattering of HW by stray dogs or cats was recognized as a major challenge in HW management (39.8%).

### Risk factors associated with biomedical waste related occupational health hazards

Occupational designation, level of training received in HW management, professional experience, vaccination status for tetanus, degree of knowledge and attitudes on HW management were recognized as the significant risk factors associated with HW related health effects (*p < 0.05*). Among HCP, attendants denoted a significantly higher susceptibility to HW related hazards (OR = 3.82; 95% Confidence Level [CI] = 2.68–4.94), followed by Nursing Officers (OR = 3.22; 95% CI = 2.23–4.17) and Minor Staff/Labourers (OR = 2.44; 95% CI = 1.68–3.40), as shown in Table 4. Respondents with higher working experience were less susceptible (*p = 0.027*) for being influenced with HW related health impacts, when compared to the respondents with work experience <3 years. Further, HCP, who had undergone trainings on HW management were characterized with a lower experiencing rate of health effects, while HCP without any training reported a significantly high prevalence (OR = 13.31; 95% CI = 11.11–14.01; *p = 0.01*). Meanwhile, HCP who had not been vaccinated for tetanus denoted a significantly higher (OR = 9.49; 95% CI = 7.58–10.11; *p = 0.02*) susceptibility to HW related hazards (Table 4). In addition, respondents who had higher knowledge levels on HW management (*p < 0.001*) also demonstrated a significantly lower likelihood of experiencing health effects due to HW. A similar trend was observed for attitude level also (*p < 0.001*).

**Table 4:** Socio-economic risk factors associated with biomedical waste related health effects.

Parameter		Total respondents	Respondents had experienced Health Impacts		*p* Value	Odds Ratio (OR)	95% Confidence Intervals (CIs)	
			n	%			Lower	Upper
Designation	Medical officer	85	6	7.1		Reference		
	Nursing officers	117	23	19.7	0.046	3.22	2.23	4.17
	Paramedical staffs	37	2	5.4	0.037	0.75	0.99	2.40
	Attendant	40	9	22.5	0.013	3.82	2.68	4.94
	Minor Staff/ Labourers	128	20	15.6	0.046	2.44	1.68	3.40
Experience	< 3 years	65	23	35.4		Reference		
	3 to 5 years	109	21	19.3	0.048	0.57	0.50	0.82
	6 to 10 years	106	10	9.4	0.037	0.46	0.17	0.75
	> 10 years	127	6	4.7	0.023	0.20	0.05	0.80
Trained in Medical Waste Management	Yes	271	11	4.1		Reference		
	No	136	49	36.0	0.01	13.31	11.11	14.01
Vaccinated for Tetanus	Yes	291	17	5.8		Reference		
	No	116	43	37.1	0.02	9.49	7.58	10.11
Knowledge on Medical Waste Management	Poor	5	4	100.0		Reference		
	Moderate	89	32	34.8	0.005	0.14	0.05	0.21
	High	313	24	7.7	0.001	0.08	0.01	0.18
Attitudes on Medical Waste Management	Moderate	219	50	22.8		Reference		
	High	188	10	5.3	<0.001	0.10	0.04	0.27

The Binary Logistic Regression (BLR) with forward step-wise variable selection method was used for the above analysis.

## Discussion

Medical waste management has become a major problem for healthcare facilities worldwide due to increasing rates of HW generation, lack of adequate proper waste management utilities and limited knowledge and training among staff (22, 23). As a developing country, Sri Lanka also faces this challenge, while information available on current situation related to HW is limited. Around 76.9% of respondents were characterized with a high knowledge level on HW management, which is better than other neighboring countries in the region such as India (24, 25), Bangladesh (9) and Northwest Ethiopia (26). Although a notable fraction of the study population (57.2%) was having more than 5 years of experience in the health sector, only 65.4% of respondents were aware of HW management. Knowledge about HW management among the technically qualified HCP (Medical officers, nurses, and paramedical staff) was found to be satisfactory, in comparison to the attendants and labourers. This agrees with the findings of several previous studies (2, 27). Since poor knowledge and training on HW management may lead into serious health consequences and detrimental impacts on the environment (28), sufficient knowledge of all HCP on HW management is important. Therefore, addressing such issues is a timely requirement.

Knowledge regarding the classification system of HW and respective colour coding systems are highly important aspects in HW management. Present study revealed that the majority of respondents were familiar with the HW based colour coding system in Sri Lanka. A similar finding has been reported in Bangalore, where 96.1% of respondents had been aware of the colour coding system of HW (28). In addition, a higher fraction of respondents of this study were familiar with the biohazard symbol, which is also similar to the aforesaid study. Further, 72.2% of respondents were familiar with the maximum storage time of HW, which was significantly higher than in Ethiopia (26). Meanwhile, a notable faction of respondents (66.6%) had already received previous trainings on HW management, which could be the underlying reason behind the elevated awareness level. However, only a limited faction of respondents knew that body fluids, body parts and fecal matter are considered as pathological waste. Although, general knowledge regarding HW was found to be significantly high, several knowledge gaps in certain specific areas were found. Such knowledge gaps might lead into inappropriate practices among HCP (2). Therefore, organizing routine awareness programmes to enhance the knowledge and training of HCP on HW is essential.

Although, the majority of respondents in this study valued proper segregation and management of HW (91.1%), in Nepal only 6.0% of respondents have agreed with the importance of HW segregation (29). Around 50.8% of respondents were considering such practices as an additional work burden, which is common to many developing countries. Implementing a proper mechanism for HW collection, transportation and treatment remains difficult due to financial and human resource related challenges (28, 29). Few respondents perceived that government is entirely responsible for proper disposal of HW. A previous study in Bangalore has reported that only a limited faction of respondents considered government to be entirely responsible for proper disposal of HW (28). However, the attention of administrative staff on proper HW management was reported to be low (35.6%) at the institution level, while around 63.9% of respondents were satisfied with the adequacy of HW management practices. Effective management of HW is not only a legal necessity, but also a social responsibility (29). Findings of this study revealed that a higher fraction of the respondents have cultivated such attitudes in Sri Lanka.

Present study revealed that HW segregation was being practiced at a satisfactory level, while studied hospitals were having adequate facilities and procedures for HW treatment and disposal. But a study conducted in Nepal, has reported that HW storage facilities are not adequate (28). Incineration (82.6%), sterilization (54.8%) and chemical treatment (38.3%) were recognized as the major HW treatment strategies in studied hospitals. A previous study has reported open burning in a hole (54%), low-temperature incineration (52%) and open-air burning on the ground (18%) as the major HW treatment practices in Ethiopia (26, 30). Another study from Hawassa city has reported that low combustion incinerators and open burning methods are used to treat HW in Ethiopia (31).

The majority of respondents in the current study had been vaccinated for Hepatitis B and Tetanus. This contradicted with the findings from a previous study conducted in Ethiopia, where only 20 and 40% of HCP were vaccinated for HBV and tetanus toxoid, respectively (26). Owing to the occupational hazards associated with HW, use of adequate PPE is essential to avoid unnecessary health effects. Findings revealed that around a higher fraction of respondents were using PPE, such as gloves (98.5%), boots (84.3%) and aprons (74.6%), during handling of HW. However, several previous studies conducted in Northwest Ethiopia (26) and South Africa (32) have reported very lower rates of PPE usage. Moreover, in Southeast Nigeria and Tanzania, less than one-third (30%) of HCP had been provided with PPE and access to prophylaxis to avoid any health hazards arising from improper HW management (33, 34). Not being vaccinated for tetanus was also found as a significant risk factor. Being vaccinated for potential health effects such as tetanus, is a timely precaution, which could slim down the chances of facing severe health hazards associated with improper HW management. According to a study conducted in South Africa, a notable fraction of HCP was handling HW with their bare hands due to shortages in gloves, regardless of the provisions of the occupation Health and Safety Act in South Africa (32). This clearly suggests that despite the availability of regulations and national health policies concerning the HW management, a limited attention is placed on HW management in many countries (35).

A risk perception analysis conducted in Portugal has revealed that doctors and nurses in general show a higher risk perception than general staff (16). Better awareness levels on risks associated with HW among higher occupational categories has been identified as the major reasons for this (16). The limited access to information on proper HW management among lower occupational categories of HCP had further aggravated this. Respondents with higher working experience, knowledge and proper training on HW management were less susceptible for being influenced with HW related health hazards. Higher service period in the health sector empowers HCP to gain more experience and knowledge regarding HW management, thereby enabling them to handle HW with care based on standard guidelines (32, 34).

A study conducted in Jamaica (34) has revealed that the majority of HCP (98.1%), despite being doctors or nurses, had insufficient knowledge on HW management. A similar trend has been observed in India (36) and Bangladesh (37). However, 76.9% of respondents of this study were characterized with an appropriate knowledge on standard HW management, which is highly satisfactory compared with neighboring countries. A higher risk of infectious disease transmission is faced by HCP at the global level, especially in low-income countries, due to improper handling of HW (8, 38, 39). Meanwhile, Anozie et al. (40) has emphasized that the risk of occupational exposure to HW related hazards is worse in developing countries. In many developing countries, HW has not received the much-needed attention that it deserves (41–43), mostly due to inadequate resources and awareness, making it a low priority (44). However, in the present study, a notable level of respondents was satisfied with the contingency plans available for management of infectious HW during a sudden disaster (54.3%) and regular inspection and periodic maintenance programmes on HW management (57.5%).

Adequate level of awareness and training on HW management directly influence the perceptions and practices of HCP (34). Unsatisfactory HW management practices among HCP and limited availability of properly trained manpower to handle HW are major challenges faced by healthcare institutions in developing countries (45). Deficiencies in financial resources, required infrastructure and other facilities have further aggravated this situation. Therefore, implementing routine training programmes for HCP regarding the proper HW is critical to reduce occupational hazards. Further, strengthening the existing regulation framework, enhancing the motivation and commitment of administrators of healthcare institutions and provision of adequate level of financial and human resources are also important to increase the efficiency of HW management.

The current study focused only on a selected number of private and government hospitals in the Colombo District of Sri Lanka, which could be identified as a limitation. The restrictions in financial resources, time and acquiring administrative approval were behind this limitation. However, a satisfactory number of HCP were recruited from different occupational and healthcare institutional categories to compensate for the above limitation.

## Conclusion

The majority of respondents were characterized with a high knowledge level (76.9%) and positive attitudes (53.8%) on HW management. Incineration was recognized as the most widely used HW treatment method. In addition, most of the respondents were using PPE at a satisfactory level and handling HW based on standard guidelines. However, limitations were reported in routine inspection of HW management process and treatment of liquid waste. Organizing of routine training programmes was also limited. Occupational designation, level of training received in HW management, professional experience, vaccination status for tetanus, degree of knowledge and attitudes on HW management were recognized as significant risk factors associated with HW related occupational hazards.

Compared to other developing countries in the region, treatment of HW in Sri Lanka was satisfactory. However, more attention should be placed on existing regulations on HW management. Strengthening the existing mechanisms for continuous monitoring of HW management and provision of more resources (financial, human and technological) are recommended to enhance the efficiency of HW management in Sri Lanka. In addition, administrative officers should be motivated to support the HW management processes at the institutional level and a proper framework should be developed to organize training and awareness programmes on HW management for HCP.

## Data availability statement

The original contributions presented in the study are included in the article/Supplementary material, further inquiries can be directed to the corresponding author.

## Ethics statement

Ethical approval was obtained from the Ethics Review Committee (ERC) of the National Institute of Health Science (NIHS), Kaluthara, Sri Lanka (ECR Clearance No: NIHS/ERC/21/05RR). Permission from Regional Director of Health Service, Colombo District and respective Director and Superintendents of each hospital was obtained, prior collection of data. The written informed consent was obtained from all the participants for participating prior to conducting the survey. The confidentiality of the acquired data was maintained throughout the study. The entire study was conducted adhering to regulations and guidelines of the ERC.

## Author contributions

LU: conceptualization. LS and AP: data curation. LU: formal analysis. LU, AP, and LS: investigation, methodology. LU and KR: writing – original draft. TR: writing – review & editing. All authors contributed to the article and approved the submitted version.

## Abbreviations

BLR: Binary Logistic Regression
HW: Healthcare Waste
HCP: Healthcare Professionals
LKR: Sri Lankan Rupees
MCQ: Multiple-Choice Questions
OR: Odds Ratio
PDHS: Provincial Department of Health Services
PPE: Personal Protective Equipment
USD: United States Dollars
WHO: World Health Organization
