# Supplementary material for: Knowledge, perceptions and practices on healthcare waste management and associated occupational health hazards among healthcare professionals in the Colombo District, Sri Lanka: a cross-sectional study
Source: Front Public Health. 2023 Dec 27;11:1215648. doi: 10.3389/fpubh.2023.1215648 (PMC10786667; doi:10.3389/fpubh.2023.1215648)
Supplement: Supplementary file 1 [file Table_1.DOCX]

**Table S1:** Practices on Biomedical Waste Management

| Parameter | | Total respondents | |
| --- | --- | --- | --- |
|  |  | **n** | **%** |
| What treatment method/s is/are used for HW management in your facility? | Chemical treatment | 156 | 38.3 |
|  | Handover to the municipal | 107 | 26.2 |
|  | Incineration | 336 | 82.6 |
|  | Pit burial | 57 | 14.0 |
|  | Sanitary landfill | 87 | 21.4 |
|  | Sterilization | 223 | 54.8 |
|  |  |  |  |
| If burning is practiced, health care waste is incinerated in, | A hole | 53 | 13.0 |
|  | A Pit/Special oven | 13 | 3.2 |
|  | Low temperature incineration | 99 | 24.3 |
|  | Open air burning on the ground | 239 | 58.7 |
|  | Open burning on the ground | 3 | 0.7 |
|  | | | |
| If an incinerator is used, is it fenced to prevent unauthorized access? | Yes | 294 | 72.2 |
|  | No | 113 | 27.8 |
|  | | | |
| Do you/waste handlers use personal protective equipment during handling the waste? | Yes | 348 | 85.5 |
|  | No | 59 | 14.5 |
|  | | | |
| What type of protective equipment do you use? | Gloves | 401 | 98.5 |
|  | Boots | 343 | 84.3 |
|  | Aprons | 304 | 74.6 |
|  | Goggles | 150 | 36.8 |
|  | | | |
| How often protective equipment are used in the handling of health care waste by waste handlers? | Always | 244 | 60.0 |
|  | I don’t know | 38 | 9.3 |
|  | Occasionally | 58 | 14.3 |
|  | Sometimes | 67 | 16.5 |
|  | | | |
| How many times per day waste is collected at your hospital? | Once | 54 | 13.3 |
|  | Twice | 153 | 37.6 |
|  | Thrice | 157 | 38.6 |
|  | More than 3 times | 43 | 10.6 |
|  | | | |
| Human anatomical waste/ animal wastes are treated or disposed through, | Deep burial | 176 | 43.2 |
|  | Disinfection | 89 | 21.8 |
|  | Incineration | 289 | 71.0 |
|  | Local autoclaving | 104 | 25.5 |
|  | Micro-waving | 91 | 22.3 |
|  | | | |
| Microbiology/ biotechnology waste is treated or disposed through, | Deep burial | 102 | 25.0 |
|  | Disinfection | 110 | 27.0 |
|  | Incineration | 253 | 62.1 |
|  | Local autoclaving | 105 | 27.7 |
|  | Micro-waving | 91 | 22.3 |
|  | | | |
| Sharps wastes are treated or disposed through, | Deep burial | 106 | 26.0 |
|  | Disinfection | 96 | 23.5 |
|  | Incineration | 241 | 59.2 |
|  | Local autoclaving | 78 | 19.1 |
|  | Microwaving | 111 | 27.2 |
|  | | | |
| Disposal method used for expired drugs, | Burning | 41 | 10.1 |
|  | I don’t know | 87 | 21.4 |
|  | Return to national medical stores | 196 | 48.2 |
|  | Disposal through another agent | 83 | 20.4 |
|  | | | |
| Healthcare facility treats liquid waste before being released | Yes | 234 | 57.5 |
|  | No | 173 | 42.5 |
|  | | | |
| Relevant staff handling medical waste is given a training on medical waste management | Never | 5 | 1.2 |
|  | Occasionally/rarely | 116 | 28.5 |
|  | Routinely | 165 | 40.5 |
|  | When requested | 121 | 29.7 |
|  | | | |
| Waste is properly segregated at the source according to different categories | Yes | 360 | 88.5 |
|  | No | 47 | 11.5 |
|  | | | |
| Waste collection bins are cleaned with appropriate disinfectants (0.5% sodium hypo chloride) daily | Yes | 249 | 61.2 |
|  | No | 158 | 38.8 |
|  | | | |
| Sharps waste are collected to puncture proof boxes | Yes | 371 | 91.2 |
|  | No | 36 | 8.8 |
|  | | | |
| Used syringe needles are collecting without recapping | Yes | 325 | 79.9 |
|  | No | 82 | 20.1 |
|  | | | |
| Infectious medical wastes are collected from service areas within 24 hours | Yes | 321 | 78.9 |
|  | No | 86 | 21.1 |
|  | | | |
| Medical waste is always transported in closed containers | Yes | 346 | 85.0 |
|  | No | 61 | 15.0 |
|  | | | |
| Plastic bags are used for non-sharps infectious waste of good quality or specialized reusable containers are used | Yes | 301 | 74.0 |
|  | No | 106 | 26.0 |
|  | | | |
| Healthcare facility has a plan for treatment and disposal of hazardous chemical, pharmaceutical and radioactive waste | Yes | 278 | 68.3 |
|  | No | 129 | 31.7 |
|  | | | |
| Incineration ash is disposed to a municipal landfill or openly dumped | Yes | 196 | 48.2 |
|  | No | 211 | 51.8 |
|  | | | |
| Storage facilities meet the proper requirement | Yes | 262 | 64.4 |
|  | No | 145 | 35.6 |
|  | | | |
| Laboratory cultures and stock of infectious agents are treated within healthcare facility before being taken away from the facility | Yes | 217 | 53.3 |
|  | No | 190 | 46.7 |
|  | | | |
| Contingency plans are there for treatment of infectious waste in the event that the treatment facility is shut down for repair | Yes | 221 | 54.3 |
|  | No | 186 | 45.7 |
|  | | | |
| Healthcare facility has a programme of regular inspection and periodic maintenance of the treatment facility | Yes | 234 | 57.5 |
|  | No | 173 | 42.5 |
|  | | | |
| Healthcare facility uses an approved treatment technology for treating medical waste | Yes | 245 | 60.2 |
|  | No | 162 | 39.8 |
|  | | | |
| Stray dogs or cats in the hospital premises sometimes scatter collected/stored waste | Yes | 162 | 39.8 |
|  | No | 245 | 60.2 |
